# Prevalence of Diabetes and Its Determinants in the Young Adults Indian Population-Call for Yoga Intervention

**DOI:** 10.3389/fendo.2020.507064

**Published:** 2020-12-11

**Authors:** Raghuram Nagarathna, Parul Bali, Akshay Anand, Vinod Srivastava, Suchitra Patil, Guruprasad Sharma, Krishna Manasa, Viraaj Pannu, Amit Singh, Hongasandra R. Nagendra

**Affiliations:** ^1^ Vivekananda Yoga Anusandhana Samsthana, Bengaluru, India; ^2^ Department of Biophysics, Postgraduate Institute of Medical Education and Research, Chandigarh, India; ^3^ Neuroscience Research Lab, Department of Neurology, Postgraduate Institute of Medical Education and Research, Chandigarh, India; ^4^ College of Social Work, University of Kentucky, Lexington, KY, United States; ^5^ Department of Yoga and Life Science, Swami Vivekananda Yoga Anusandhana Samsthana, Bengaluru, India; ^6^ Government Medical College and Hospital Sector 32, Chandigarh, India

**Keywords:** prevalence, diabetes, young adult Indian population, IDRs, lifestyle - related disease

## Abstract

**Background:**

The young Indian population, which constitutes 65% of the country, is fast adapting to a new lifestyle, which was not known earlier. They are at a high risk of the increasing burden of diabetes and associated complications. The new evolving lifestyle is not only affecting people’s health but also mounting the monetary burden on a developing country such as India.

**Aim:**

We aimed to collect information regarding the prevalence of risk of diabetes in young adults (<35 years) in the 29 most populous states and union territories (7 zones) of India, using a validated questionnaire.

**Methods:**

A user-friendly questionnaire-based survey using a mobile application was conducted on all adults in the 29 most populous states/union territories of India, after obtaining ethical clearance for the study. Here, we report the estimation of the prevalence of the risk of diabetes and self-reported diabetes on 58,821 young individuals below the age of 35 years. Risk for diabetes was assessed using a standardized instrument, the Indian diabetes risk score (IDRS), that has 4 factors (age, family history of diabetes, waist circumference, and physical activity). Spearman’s correlation coefficient was used to check the correlations.

**Results:**

The prevalence of high (IDRS score > 60), moderate (IDRS score 30–50), and low (IDRS < 30) diabetes risk in young adults (<35 years) was 10.2%, 33.1%, and 56.7%, respectively. Those with high-risk scores were highest (14.4%) in the Jammu zone and lowest (4.1%) in the central zone. The prevalence of self-reported diabetes was 1.8% with a small difference between men (1.7%) and women (1.9%), and the highest (8.4%) in those with a parental history of diabetes. The south zone had the highest (2.5%), and the north west zone had the lowest (4.4%) prevalence.

**Conclusions:**

Indian youth are at high risk for diabetes, which calls for an urgent action plan through intensive efforts to promote lifestyle behavior modifications during the pandemics of both communicable and noncommunicable diseases.

## Introduction

India is a fast developing economy with a considerable number of diabetes patients. Its health care cost is rising with a deterioration in health standards among the economic productive young population (1–4). It is the country with the second highest numbers after China with 65.1 million diabetes cases that estimated in 2013. This is expected to increase up to 109.0 million in 2035 (5). The highest prevalence of diabetes was noted in low-income countries (LIC) and lowest in high-income countries (HIC) (6). The diabetes primarily affects individuals over 50 years of age in HIC, whereas in middle-income countries (MIC), the prevalence is higher in young individuals, which is the most productive age group. The prevalence in older age again rises as these young individuals age with increased life expectancies (5, 7).

Diabetes has become a global pandemic and threat for world health due to demographic variations and cultural differences of societies supplemented by aging phenomena. It is a costly disease that has been identified as the prime causative factor for blindness, lipoprotein abnormalities, or mitochondrial dysfunction causing cardiovascular diseases, renal failure, and amputation in several countries (8–10). The World Health Organization (WHO) has reported 24 million cases of diabetic neuropathy, 5 million cases of retinopathy, and 6 million cases of amputation due to diabetes. The mortality in individuals with diabetes is chiefly due to cardiac complications. Therefore, diabetes can cause undesirable consequences and, hence, needs urgent consideration in the young population in order to timely strategize effective prevention therapies (8, 11).

Genetic and environmental factors, such as heredity, change in lifestyle, age, smoking habits, increased alcohol consumption, screen time, parental conflicts, improper sleep, education, and stress, predispose young adults to diabetes, which is exacerbated with diabetic comorbid conditions (12). Obesity is the main risk factor that accounts for 80%–85% of the risks of developing type-2 diabetes (13).

The lack of physical activity among the younger population is a matter of concern as 84% of girls and 78% of males in Australia did not meet the criteria for minimum physical activity corresponding to their age. As a consequence, females were found to be more overweight than males (14). The risk of diabetes in young adults can be managed by routine physical activity and adopting a healthy and balanced diet, which focuses on the increased intake of dietary fiber (15–17). The WHO strongly recommends reducing the intake of free sugars throughout one’s lifetime by avoiding foods or beverages containing added monosaccharides and disaccharides (18). A study was conducted in an urban slum in a large metropolitan city in northern India, which noted a high prevalence of metabolic disorders, such as obesity, dyslipidemia, and diabetes mellitus in middle age, particularly in females in such an economically deprived population (19). Hence, such prevalence studies are required even at a national level to examine the important risk factors in this economic productive young population in order to have effective prevention strategies.

Our study was aimed to estimate the prevalence of low, moderate, and high risk of diabetes in young adults. We conducted a nationwide study by collecting information regarding prevalence of risk of diabetes in young adults using a validated questionnaire. Moreover, the contribution of other sociodemographic factors, such as age, physical activities, yoga, family history, vitals, diet, gender, marriage, education, occupation, and socioeconomic status, were further collected to examine diabetic progression.

## Methods

### Sampling and Study Population

The study was conducted after ethical clearance from the ethical committee of the Indian yoga association with reference number RES/IEC-IYA/001. The data used in this analysis has been collected during phase 1 of the NMB 2017 trial, a large translational, multicenter, cluster-sampled research trial aimed to assess the efficacy of yoga-based lifestyle modification as a primary prevention strategy for diabetes in a community setting. The methodological details of the study have been reported previously (20, 21). In brief, the data collection aimed at screening 4000 adults per district in 60 randomly selected districts representative of the Indian adult population. There were two research associates (who designed the study and monitored work of senior research fellows), 30 senior research fellows (who worked in each district and monitored the work of yoga volunteers for diabetes movement [YVDMS]). The 1200 YVDMs were involved in data collection and yoga training in the next part of the study. These YVDMs were trained for data collection as per their schedule (**Supplementary Table 3**).

### Sample Size Estimation

Keeping in mind the twin objectives of the study, the sample size estimation was based on the relative risk reduction (30%) in prediabetes individuals reported in the Community Lifestyle Improvement Program study (22). We used annual incidence rates of diabetes as 18.3% in the control conditions as per IDPP-1 study (23). This provided a conversion rate at 3-month follow-up of 4.57% and 3.0%, respectively, for control and intervention conditions. Using the sample size calculator (http://www.sample-size.net), the required sample size for a two-group design with α = 0.05 and (1− α) = 0.80 was estimated to be 1949 for each group (a total of 3898 individuals). Factoring an attrition of 20%, the final sample size was estimated to be 4678 individuals with prediabetes. To obtain 4678 individuals with prediabetes, it was calculated that there was a need to screen 77,967 adults above the age of 20 years (4678 × 100/6; the least reported prevalence of prediabetes in India has been 6.0% (24). Thus, the study plan included screening of approximately 155,933 individuals across 60 Indian districts (10% of all districts as per the 2011 Census of India), assuming a nonresponse rate of 50%. Consequently, the study targeted approximately 4000 adults per district with equal involvement of the urban and rural areas.

### Assessments

We acquired information on diabetes and risk scores by a door-to-door survey using a mobile application with detailed person-level information about age, gender, income details, educational qualifications, and marital status.

The Indian Diabetes Risk Score (IDRS) developed by Mohan et al. in 2005 was used for risk analysis (25). IDRS is a validated instrument with optimum sensitivity (72.5%) and specificity (60.1%) used widely in India in several studies (26). It is a convenient, simple, and economical tool for the detection of a high-risk population that uses age, waist circumference, parental diabetes history, and physical activity (27) (**Supplementary Table 4**). The combined scores of the 4 factors contribute to the prediction of risk level of an individual. The individuals with scores > 60, 30–50, and <30 are considered to be high, moderate, and low risk, respectively (**Supplementary Table 1**). We measured waist circumference in centimeters using a measuring tape. Self-reported diabetes was confirmed by checking the medication that they were taking and/or medical reports during the door-to-door visits. The questionnaire was tested for interrater reliability in a preliminary study between two YVDMs using the Kappa coefficient value, which was found to be 0.83.

### Sampling Strategy

**Figure 1:**
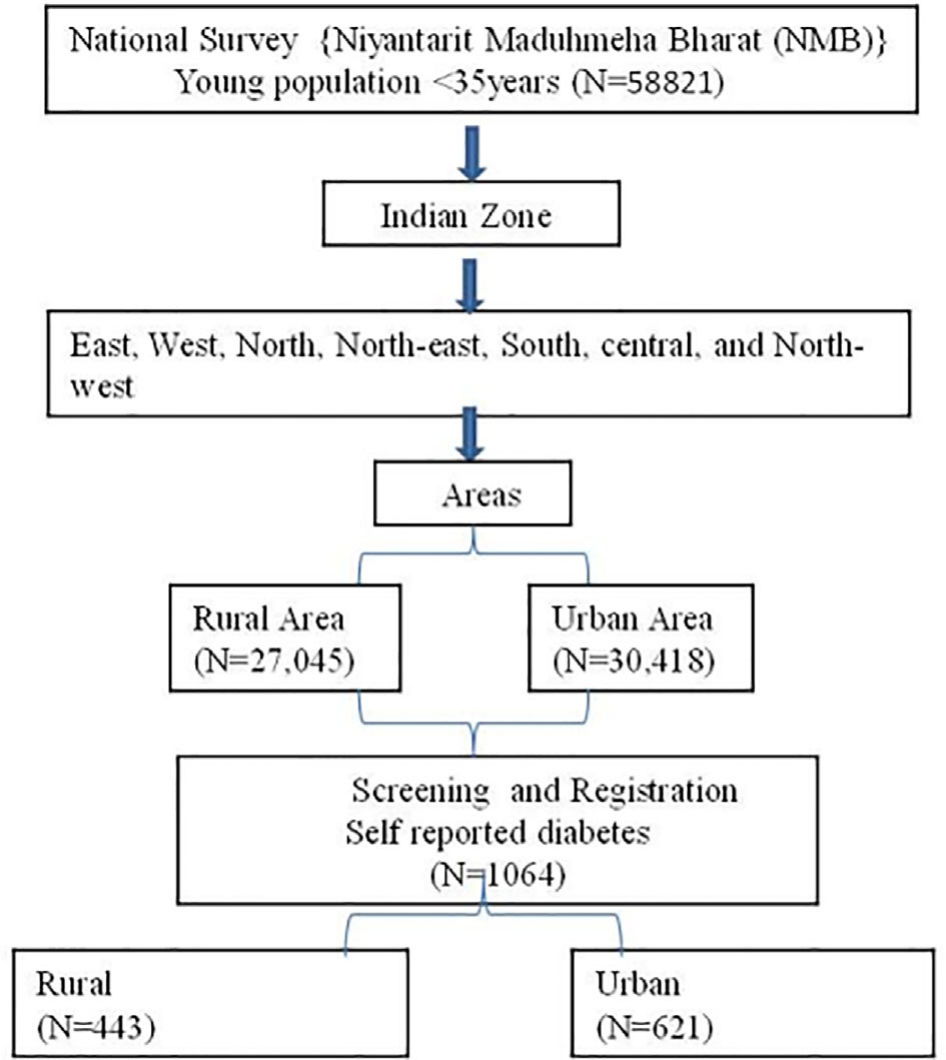
Population sampling strategy of nationwide NMB study.

*Niyantarit Maduhmeha Bharat* (NMB) 2017 was a pan-India randomized multicluster translational trial with dual objectives, namely, a survey for prevalence and lifestyle intervention for the population at high risk and known diabetes (**Figure 1**). Details of the methods have been published (20, 21) earlier. In brief, a four-stage (zone–state–district-urban/rural) strategy was adopted for identifying study locations, using a random cluster sampling method and located households and individuals. Clustering was performed by dividing each state into districts and each district into rural and urban localities. Census enumeration blocks (CEB) were randomly selected from the randomly selected wards, and all eligible individuals (both genders between 20 and 70 years) within the CEB were contacted. The door-to-door survey enlisted eligible individuals and specifically enquired about the status of diabetes and scored them on the IDRS.

Field personnel [1200 volunteers (20/district), supervised by 35 senior research officers and 5 zonal coordinators] were trained in a 5-day residential program to ask appropriate questions in local languages that included practical tests by visiting nearby villages and urban wards.

### Statistical Analysis

Data were analyzed using SPSS (21.0) version. The estimation of prevalence was calculated using the distribution of frequency and percentage using cross tabs descriptive. *Chi-square* and *Fisher exact* tests were used for mean differences. Binary logistic regression analysis was done to find the association between independent predictors of diabetes. Self-reported diabetes was considered as a dependent variable. Gender, area, marital status, parental history, IDRS, physical activity, and waist circumference were covariates by keeping the reference factors rural for area, female for gender, vegetarian for diet (**Supplementary Table 2**) etc. as mentioned in **Table 5**.

## Results

### Prevalence of Self-Reported Diabetes and Its Risks Based on Gender, Marital Status, and Parental History

According to the national survey (NMB-2017), the young diabetes population was screened across the nation on the basis of IDRS and self-reported diabetes, using validated IDRS; 60,194 individuals were selected on the basis of IDRS score, and 58,821 were selected on the basis of self-reported diabetes as young adults (<35 years). Gender-related risk of diabetes was found to be similar in men and women. The prevalence of self-reported diabetes in young females was 1.9% and in men 1.7%. On the basis of IDRS risk, no significant difference was found in the female and male diabetes population (**Table 1**). The marital status analysis revealed that 1.5% of unmarried, 2.0% of married, and 1.5% of separated individuals were found to have self-reported diabetes. Among these, married (11.6%) and separated (11.0%) individuals were under higher risk of diabetes than unmarried ones (*p* < 0.001) (**Table 1**). Similarly, the frequency distribution of unmarried, married, and separated people based in IDRS was also found to be significantly different among these groups.

**Table 1:** Frequency distribution of diabetes participants (self-reported) with context to gender, anthropometric parameters, and different geographical locations in India.

Variables		Total (%)	Diabetes(Self-reported)			IDRS							
						Total	Low Risk		Moderate Risk		High Risk		p-value
			N	%	p-value		N	%	N	%	N	%	
		58821	1078	1.8%		60194	34145	56.7%	19933	33.1%	6116	10.2%	<0.001
Gender	Male	27720	479	1.7%	0.059	28287	16137	57.0%	9324	33.0%	2826	10.0%	0.157
	Female	30708	585	1.9%		31552	17836	56.5%	10473	33.2%	3243	10.3%	
Area	Rural	27045	486	1.8%	0.221	27998	16230	58.0%	9028	32.2%	2740	9.8%	<0.001
	Urban	30418	574	1.9%		30766	17029	55.4%	10502	34.1%	3235	10.5%	
Parental history of DM	Non- parents	46521	618	1.3%	<0.001	51009	31269	61.3%	16579	32.5%	3161	6.2%	<0.001
	One parents	6653	333	5%		7317	2189	29.9%	2844	38.9%	2284	31.2%	
	Both parents	1061	89	8.4%		1136	96	8.5%	381	33.5%	659	58.0%	
Marital status	Married	40525	805	2.0%	<0.001	42166	22225	52.7%	15049	35.7%	4892	11.6%	<0.001
	Un-married	15383	236	1.5%		16097	10686	66.4%	4320	26.8%	1091	6.8%	
	Separated	65	1	1.5%		73	42	57.5%	23	31.5%	8	11.0%	
BMI	Under weight (<18.5)	3665	41	1.1%	<0.001	3884	3103	79.9%	677	17.4%	104	2.7%	<0.001
	Normal weight (18.5-25)	21797	293	1.3%		23078	15061	65.3%	6463	28.0%	1554	6.7%	
	Over- weight (25-30)	8548	201	2.4%		9031	3858	42.7%	3607	39.9%	1566	17.3%	
	Obese (>30)	3291	110	3.3%		3448	1277	37.0%	1309	38.0%	862	25.0%	
Waist circumference	High Risk	10556	338	3.2%	<0.001	11230	566	5.0%	6887	61.3%	3777	33.6%	<0.001
	Moderate risk	17025	346	2%		18706	5776	30.9%	10733	57.4%	2197	11.7%	
	Normal	26617	355	1.3%		29526	27212	92.2%	2184	7.4%	130	0.4%	
IDRS	Low- risk	30775	335	1.1%	<0.001	–							–
	Moderate risk	18442	338	1.8%									
	High- risk	5638	366	6.5%									
Physical activity	No	7274	148	2.0%	0.084	12191	4828	39.6%	4087	33.5%	3276	26.9%	<0.001
	Mild	12249	265	2.2%		23215	8582	37.0%	12434	53.6%	2199	9.5%	
	Moderate	21921	395	1.8%		16581	12979	78.3%	2983	18.0%	619	3.7%	
	Vigorous	15028	246	1.6%		8207	7756	94.5%	429	5.2%	22	0.3%	

Interestingly, it has been found that 1.3%, 5%, and 8.4% of diabetic subjects were self-reported with no parental history of diabetes, one diabetes parent, and both diabetes parents with diabetic history, respectively (*P* < 0.001). Frequency distribution based on the parental history of diabetes has also reflected significantly higher numbers in high IDRS scores as compared to low-risk IDRS. Results suggest the inheritance pattern of diabetic condition, which may be triggered with familial lifestyle or genetic susceptibility of parents and trait transmission in siblings.

### Prevalence of Self-Reported Diabetes and Its Risk Based on BMI, Physical Activity, and Waist Circumference

Participants were categorized into normal, underweight, and overweight/obese. It was found that the overweight (2.4%) and obese (>30) (3.3%) young population was at significantly higher risk of diabetes than the normal (1.3%) and underweight (1.1%) young population (**Table 1**). The percentage of self-reported diabetes individuals with normal, high, and moderate health risks based on waist circumference is as follows: 1.3%, 3.2%, 2.0% (*p*< 0.001). The IDRS scores (based on waist circumference) were also significantly higher in high-risk participants based on waist circumference of individuals, i.e., almost 33.6% more than moderate (11.7%) and normal (0.4%) individuals (**Table 1**).

The frequency distribution based on physical activities was also in concordance with the BMI and waist circumference of the participants. It was found that the proportion of individuals who performed no, mild, moderate, or vigorous physical exercise were comparable.

### Differential Frequency of Self-Reported Diabetes and Its Risk Factors Based on Different Indian Geographical Location

The zone-wise prevalence of diabetes (self-reported) was significantly different (<0.001) and reported as follows in descending order: south, north, east, northeast, central, west, and Jammu. However, no gender-wise significant differences were found (**Table 2**). Zone-wise distribution of high and moderate IDRS risk of diabetes was also reported as south, north, west, Jammu, northeast, east, and central (**Table 3**), and the data showed significant differences among these groups. However, frequency distribution of self-reported diabetes was comparable in urban areas (1.9%) and rural localities (1.8%) that showed statistically insignificant differences between the two (*p* = 0.221, **Table 1**). The proportion of individuals taking treatment to control diabetes was estimated. Results demonstrated that only 54.5% of the young diabetes adults were taking treatment to control diabetes, and there were no medications being taken by 45.5% of the diabetes subjects (**Table 4**).

**Table 2:** Zone-wise frequency distribution of self-reported diabetes participants.

Zone	Total (N)	Diabetes (%)	p-value	Gender	Total (N)	Diabetes		p-value
						n	%	
North	6565	117 (1.8%)	<0.001	Male	2857	45	1.6%	=0.242
				Female	3615	71	2.0%	
South	15734	389 (2.5%)		Male	7465	182	2.4%	=0.971
				Female	8050	197	2.4%	
East	8611	152 (1.8%)		Male	3901	60	1.5%	=0.168
				Female	4663	90	1.9%	
West	8812	135 (1.5%)		Male	4564	64	1.4%	=0.296
				Female	4235	71	1.7%	
Central	8889	151 (1.7%)		Male	4148	67	1.6%	=0.555
				Female	4725	84	1.8%	
North-West	4857	44 (0.9%)		Male	2095	15	0.7%	=0.224
				Female	2762	29	1.0%	
North-East	5353	90 (1.7%)		Male	2690	46	1.7%	=0.792
				Female	2658	43	1.6%	

**Table 3:** Zone-wise risk of diabetes based on IDRS score.

		Zone							p-value
		North (n = 6844)	South (n = 12317)	East (n = 9650)	West (n = 9507)	Central (n = 10294)	Jammu (n = 5370)	North East (n = 6212)	
**IDRS**	**High risk**	848 (12.4%)	1699 (13.8%)	727 (7.5%)	1126 (11.8%)	425 (4.1%)	774 (14.4%)	517 (8.3%)	<0.001
	**Moderate risk**	2312 (33.8%)	5978 (48.5%)	2919 (30.2%)	3174 (33.4%)	1607 (15.6%)	2114 (39.4%)	1829 (29.4%)	
	**Low risk**	3684 (53.8%)	4640 (37.7%)	6004 (62.2%)	5207 (54.8%)	8262 (80.3%)	2482 (46.2%)	3866 (62.2%)	

The proportion of high, moderate, and low risks based on IDRS was calculated zone-wise that includes north, south, east, west, central, Jammu, and north-east India.

**Table 4:** Proportion of self-reported diabetes individual prescribed for treatment.

	Treatment	No treatment
**Diabetes Subjects**	54.5%	45.5%

### Relative Risk of Diabetes

By using logistic regression, high- and moderate-risk young adults (based on IDRS) were found to have higher odds of developing diabetes as compared to low-risk young adults. Unmarried young adults had 1.290 higher odds (*p* < 0.001) of diabetes as compared to married individuals. The comparison was made for relative risk of diabetes (28) within each parameter using a binary multinomial logistic regression analysis. Both higher and lower odds of diabetes as compared to the reference variable have been reproduced in **Table 5**. Logistic regression analysis to see the impact of BMI and food habits on IDRS scoring has revealed the imperative impact of both on diabetes. Obese participants can significantly stimulate the diabetic condition (**Table 6**).

**Table 5:** Multinomial logistic regression analysis showing the odds of diabetes within each variable.

Variable	Reference Variable	Dependent variable with self reported diabetes	Odds Ratio (95% CI)	p-value
Area	Rural	Urban	1.364(1.206-1.542)	0.000
Gender	Male	Female	1.197(1.061-1.351)	0.003
Parental DM history	Both non Diabetes	One parent diabetic	3.893(3.399-4.459)	<0.001
		Two parent diabetic	6.633(5.266-8.355)	<0.001
Marital status	Unmarried	Married	1.290(1.116-1.491)	0.000
Diet	Vegetarian	Non-vegetarian	2.208(0.757-5.488)	0.159
Yoga practice	No	Yes	1.613(1.332-1.954)	<0.001
IDRS	Low risk	High risk	6.211(5.340-7.223)	<0.001
		Moderate risk	1.674(1.438-1.950)	<0.001
Physical activity	Vigorous	Moderate	0.801(0.852-0.984)	0.035
		Mild	0.894(0.738-1.088)	0.203
		No	1.065(0.869-1.305)	0.548
Waist circumference	Normal risk	Moderate risk	1.535(1.322-1.782)	<0.001
		High risk	2.447(2.105-2.845)	<0.001
BMI	Underweight	Overweight	2.128(1.518-2.985)	0.000
		Obese	3.057(2.129-4.389)	0.000

The odds ratio was calculated for geographical location, gender, marital status, parental DM history, diet, yoga practice, IDRS, physical activity, waist circumference, and BMI.

**Table 6:** Logistic regression to see the association of BMI and food habit with IDRS scores.

		Variables in the Equation							
		B	S.E.	Wald	df	P-value	OR	95% C.I.	
								Lower	Upper
Step 1^a^	BMI			24.097	2	0.000			
	BMI (<23.5, Normal)	0.486	0.206	5.541	1	0.019	1.626	1.085	2.436
	BMI (23.6-27.5, obese)	0.823	0.168	24.089	1	<0.0001	2.277	1.639	3.163
	Non-veg	0.849	0.508	2.792	1	0.095	2.338	.863	6.330
	Constant	-5.305	0.518	104.737	1	0.000	0.005		

## Discussion

India is the second-largest populated country in the world. It is estimated that India has more of a young population compared to other countries in the world. According to the 2011 census, out of the total population, about 65% of the population of India are under the age of 35 (29). India has more than 40 million diabetes cases with a good majority across the nation not aware of the disease and comorbid factors. As diabetes risk varies with increasing age, early detection and intervention may prevent serious health complications and healthcare-related cost. The diabetes population in young adults has a tendency to become readily or more vulnerable to comorbid diabetes illnesses (30). Complications related to diabetes are becoming a major cause of morbidity and mortality in the young population (31). Rapidly increasing burden of Diabetes in the young might reder population to early predisposition to age related disorders which have no treatment (32–35). Primarily, the risk of diabetes is associated with age, obesity, parental diabetes history, smoking, type of diet, and physical inactivity (36).

The studies have shown that diabetes might be linked to genetic and environmental factors (37). Parental history is generally believed to play a major role in the prediction of diabetes. Therefore, we analyzed the percentage prevalence of self-reported diabetes in both parents with diabetes, no parents with diabetes, and one parent diabetes. This survey revealed that, overall, 1.3% of the diabetes cases had no parental history, which is possibly explained by the change in lifestyle or some epigenetic factors that can contribute to the development of such diabetes cases. Our study demonstrates that young adults with both diabetes and one diabetes parent are at a high risk of developing diabetes as compared to both nondiabetes parents. Comparison of the relative risk of diabetes within each variable showed significant results except gender. We observe that marital status (separated vs. married) was also found to be associated with diabetes risk. The current study suggests that unmarried individuals are also at increased risk of diabetes but less than married and separated people. This could be possibly because of more stress or hormonal changes in unmarried as compared to married people, which may be the contributing factors for developing diabetes risk; however, further studies are required to conclude these possibilities. Although, it is difficult to speculate why unmarried individuals as compared to separate and married were more affected by diabetes, it is possible that the former group ignored health and wellness as compared to the latter.

It was also found that the risk of diabetes varies according to areas and zones. Based on the IDRS score, the study found that the urban young population is under higher risk of diabetes than the rural counterparts. The southern region was found to have more young diabetes population i.e., 2.5%. The study conducted in India shows a similar prevalence of diabetes in the urban population (24). Nevertheless, the distribution characters in all cities were found to be comparable except socioeconomic status.

Dietary habits played a vital role in enhancing the diabetes risk and awareness, and more attention is required regarding this aspect. Diet, with high glycemic load, results in diabetes complications (38). Interestingly, the study outcomes reveal that the young vegetarian population was under a higher risk of diabetes than the nonvegetarian self-reported diabetes population. This reflects the predominant consumption of vegetarian diets rich in carbohydrates, such as rice, wheat, oil, and fatty foods. Additionally, it is worth noting that consumption of sweets is also an integral part and parcel of the Indian culture, which could be responsible for the development of diabetes among the young adult population (39). However, other studies suggest that the typical vegetarian diet helps in reducing the diabetes risk (40). This controversial fact needs further investigation, including the amount and types of diet with an appropriate control group. India is the habitat of different religions and many cultures having different eating behaviors and unique lifestyles. Hence, these variations, cultural diversity, customs, and heterogeneity across the nation are great challenges to associate it with diabetes even though it has been shown that changes in the dietary pattern may reduce the chance of diabetes (41).

The individuals who showed high IDRS but did not develop diabetes need to be followed up for any late development of diabetes, especially if it had not manifested in early life (< 35 years). There is a need to develop a cost-effective and preventive management program to reduce or prevent diabetes complications in young adults. As *yoga* is emerging as a cost-effective lifestyle intervention and alternative, its efficacy in the prevention of diabetes can be examined in Indian population studies where its acceptability is high. The level of physical activity index among young adults with diabetes shows that 26.9% of the young adults with high-risk diabetes did not perform any physical activity, and 9.5% and 3.7% of these individuals were engaged in mild and moderate physical activity, respectively, indicating that a sedentary lifestyle is one of the major risk factors in the development of diabetes among younger adults. Results demonstrated that only 54.5% of the young diabetes adults were taking treatment to control diabetes, and there were no medications being taken by 45.5% of the diabetes subjects (**Table 4**). The possible reason can be that patients might be asymptomatic as we analyzed in young population.

**Figure 2:**
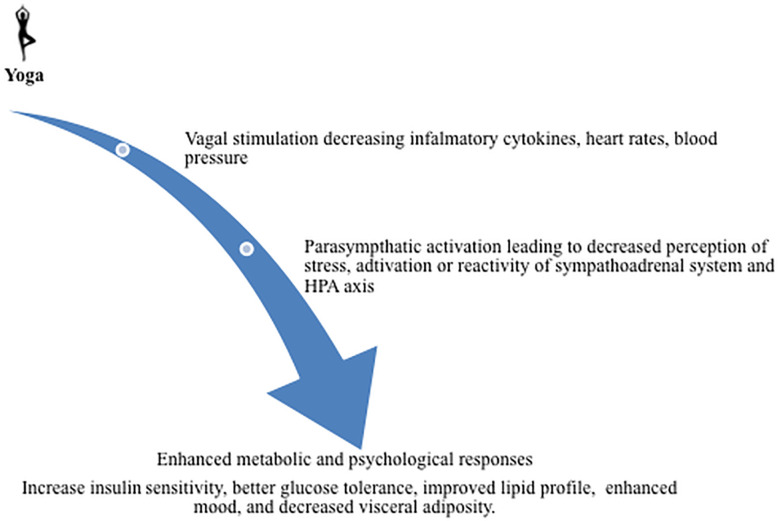
Yoga benefit in decreasing diabetic risks: The studies show that yoga causes vagal stimulation and, therefore, decreases inflammatory cytokines and heart rate as well as blood pressure. The yoga activates parasympthatic system that possibly leads to decreased perception of stress, activation, or reactivity of the sympathoadrenal system and HPA axis. Further, it may enhance metabolic and psychological responses, insulin sensitivity, glucose tolerance, improved lipid profile, mood, and decreased visceral adiposity.

Studies show that yoga helps in the activation of the hypothalamic pituitary axis and sympatho-adrenal component known to inhibit glucose uptake by inhibiting insulin release, inducing insulin resistance and increasing hepatic glucose production (42). Vigorous exercises have shown to increase HDL level, and moderate intensity exercises are effective in reducing VLDL (43). Young adults with higher risk for diabetes may benefit from practicing yoga as well as managing their obesity by engaging in vigorous and moderate intensity exercises to manage their lipid profile (**Figure 2**).

Interestingly, young diabetes patients are amenable to reversal by intensive lifestyle intervention as seen in this young diabetes study (44). The diabetes young population has greater chances of reversal because of reduced risk factors as compared to the aged group. Diabetes, if it remains untreated/undetected in the early stage of life, may become more complicated in the later stage of life (30). Young diabetes often remains undetected as aged people continue to be tested for multiple health problems and identification and corresponding intervention programs are essential for this population. This study suggests that about one fourth of the young adult population in India is at a high risk of developing diabetes and in need of the public provision of lifestyle modification programs.

### Limitations

The study used cluster sampling, which might have contributed to the sample selection bias. As a result, some subjects with diabetes might have refused to admit to having diabetes. It is also possible that a few subjects are wrongly believed to have diabetes, and there is no validation of such self-reported diabetes. Furthermore, undiagnosed diabetes could be another confounder. Subjects frequently ignore the subtle signs and symptoms of asymptomatic diabetes. The possibility of underestimation of the prevalence of diabetes in the proposed population may be the main limitation.

## Data Availability Statement

All datasets generated for this study are included in the article/**Supplementary Material**. Data is available with the principal investigator.

## Ethics Statement

Ethical permission obtained from Institutional Ethics Committee (IEC) meeting held at Indian Yoga Association, Morarji Desai National Institute of Yoga with reference no. RES/IEC-IYA/001 dated 16th Dec 2016.

## Author Contributions

RN is a grant PI involved in conceptualization, editing of manuscript. PB was involved in original writing and data analysis. VS edited the manuscript. AA was involved in conceptualization of manuscript. VS edited the manuscript. SP was involved in data curation and analysis. GS and AS were involved in the acquisition of data. VP was involved in writing, editing and collection of data on site as physician. HRN was involved in conceptualization of manuscript, obtained resources and mentoring of work. All authors contributed to the article and approved the submitted version.

## Conflict of Interest

The authors declare that the research was conducted in the absence of any commercial or financial relationships that could be construed as a potential conflict of interest.

## Supplementary Material

Supplementary Table 1 Assessments phase.

Supplementary Table 2 Diet information.

Supplementary Table 3 Schedule of 5-day training camps of Yoga-Certified Volunteers for Diabetes Movement in different zones.

Supplementary Table 4 for Physical activity measurement.
