# Supplementary material for: Prevalence of Diabetes and Its Determinants in the Young Adults Indian Population-Call for Yoga Intervention
Source: Front Endocrinol (Lausanne). 2020 Dec 11;11:507064. doi: 10.3389/fendo.2020.507064 (PMC7759624; doi:10.3389/fendo.2020.507064)
Supplement: Supplementary Table 1 — Assessments phase. [file DataSheet_1.docx]

Appendix


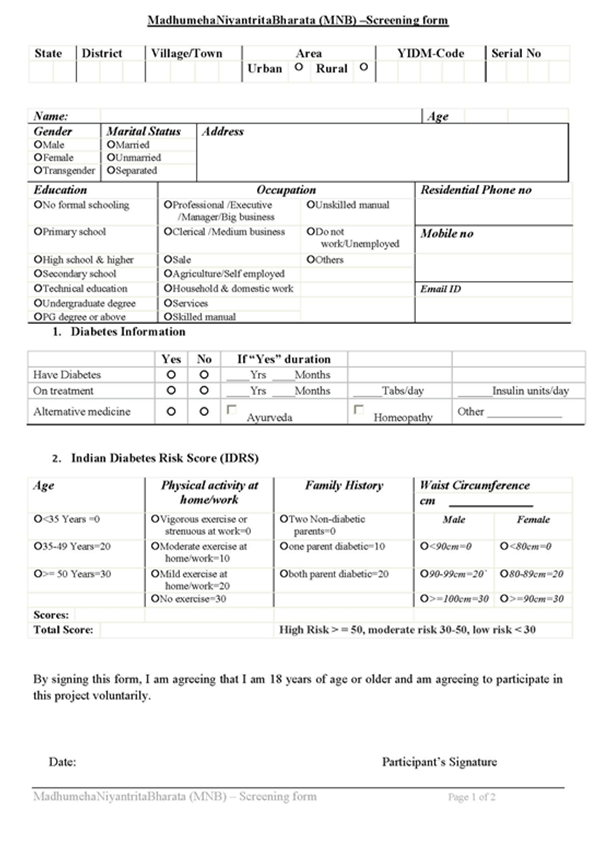


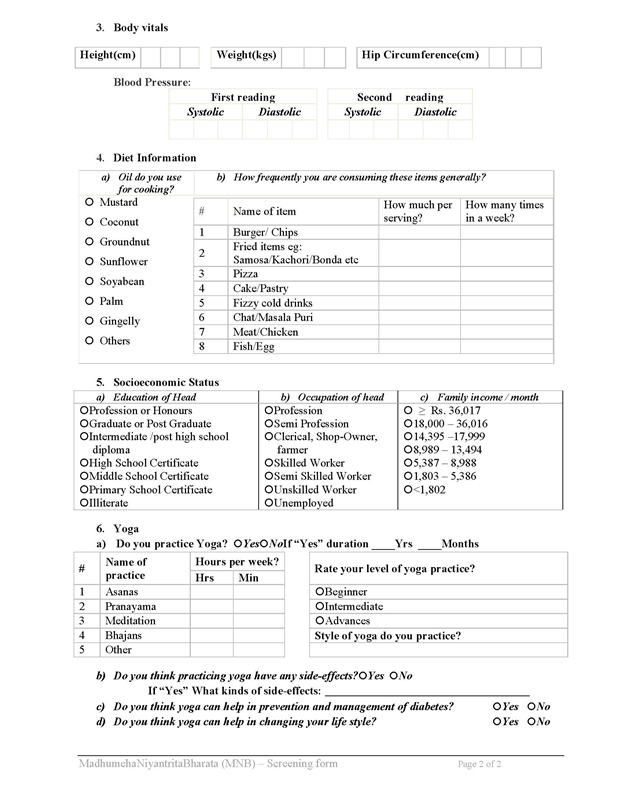


**Supplementary Table 1: Assessments phase**

| **Assessment variable** | **Method** | **Instrument used** | **Validation** |
| --- | --- | --- | --- |
| Weight | In kg | Digital weighing scale | KRUPS co. 2016 |
| Pre and post  Waist circumference | In cm | Measuring tape | Retd. Design no. 161856 |
| Pre and post  Blood pressure | MmHg | Digital sphygmomanometer | Omron co. 2016  Model HEM7120 |
| Questionnaires | Details in app  Income and expenditure | SES  Kuppuswamy’s SES scale revised for 2014  Gururaj and Maheshwaran, 2014 Monthly for, individual and family | Monthly for, individual and family  Int J Recent Trends Sci Technol 2014;11:1‑2 |
| Sleep | Sleep | Quantity, quality, and sleep routine Pre‑post | Prepared for the purpose by our team |
| Quality of life | PHQ | PHQ |  |
| Stress | PSS | 6 NAS for work, family, health, others, social and financial related stress | NAS |
| Physical activity | Typical daily work/home activity level, frequency and amount of mild, moderate, and vigorous activities |  |  |
| Substance abuse | Alcohol ‑ quantity, frequency and duration  Tobacco- Smokeless and smoked |  |  |
| Yoga related Yoga awareness | Yoga benefit scale  (Nayak *et al*. 2014) Modified  for the present Indian study[24] |  |  |

Supplementary Table 2: Diet information

| 1. ***Oil do you use for cooking?*** | 1. ***How frequently you are consuming these items generally?*** |
| --- | --- |
| \| # \| Name of item \| How much per serving? \| How many times in a week? \| \| --- \| --- \| --- \| --- \| \| 1 \| Burger/ Chips \|  \|  \| \| 2 \| Fried items eg: Samosa/Kachori/Bonda etc \|  \|  \| \| 3 \| Pizza \|  \|  \| \| 4 \| Cake/Pastry \|  \|  \| \| 5 \| Fizzy cold drinks \|  \|  \| \| 6 \| Chat/Masala Puri \|  \|  \| \| 7 \| Meat/Chicken \|  \|  \| \| 8 \| Fish/Egg \|  \|  \|   ⭘ Mustard  ⭘ Coconut  ⭘ Groundnut  ⭘ Sunflower  ⭘ Soyabean  ⭘ Palm  ⭘ Gingelly  ⭘ Others | |

**Supplementary Table 3:** Schedule of 5-day training camps of Yoga-Certified Volunteers for Diabetes Movement in different zones

| **Time zones** | **Day 1** | **Day 2** | **Day 3** | **Day 4** | **Day 5** |
| --- | --- | --- | --- | --- | --- |
| 6‑7.30 am | Arrival to  Training center | Yoga Practical Class | Yoga Practical Class | Yoga Practical Class | Yoga Practical Class |
| 7.30‑9 am | Bath and breakfast | Bath and breakfast | Bath and breakfast | Bath and breakfast | Bath and breakfast |
| 9‑10 am |  | Introduction to diabetes, medical perspectives and panchakosha level of yogic management of diabetes | Pranayama theory, practice and practical | Training for mobile apps | Examination on use mobile apps |
| 10‑11 am |  | Stress and diabetes concept and techniques of stress management through Yoga | Training for mobile apps | Individual and group  Practical examination | Examination on use mobile apps |
| 11.15‑12pm | Inauguration of  program | Yoga and modern concepts of diet for diabetes | Training for mobile apps | Practical examination |  |
| 12‑1pm | Introduction to  project | Practical chair Yoga practice | Chair Yoga Practice 2 | Practical Yoga asana examination | Valedictory program |
| 1‑2 pm | Lunch | Lunch | Lunch | Lunch | Lunch |
| 2‑3 pm | Details of project and duties of YVDM | Theory and practice of  cyclic mediation | Organization and conducting yoga camps for diabetes  Data documentation  registration from | Pranayama  examination  Cm examination | Departure |
| 3-4pm | Introduction to  cyclic meditation | Data documentation  Screening form | Data documentation  registration from | Cm examination |  |
| 4-5:30pm | Yoga practical  and CM | Screening form data taking Data documentation  registration form | Data documentation  registration from |  |  |
| 6-7:00pm | Theory and practical emotion culture and jnana yoga | Theory and practical emotion culture | Theory and practical jnana, karma, and devotion yoga for yogic stress management | Theory and practical jnana, karma, and devotion yoga for yogic stress management | Theory and practical jnana, karma, and devotion yoga for yogic stress management |

Supplementary Table 4 for Physical activity measurement

| P | **Physical activity:**  **I am going to ask you some questions about your physical activity during last one month**.  **In the last month** | |  |
| --- | --- | --- | --- |
| PMI1 | How many days do you go for a mild activity that causes no increases in breathing or heart rate like walk at a slow or normal pace? | None 0  Once a month 1  2 to 3 times a month 2  Once a week 3  2 to 3 times a week 4  4 to 5 times a week 5  Every day 6 | |
| PMI2 | On average, how many minutes of mild activity each day? | None 0 at least 10 mins 1  10 - 30mins 2  30mins - 1hr 3  1hr - 1.5hrs 4  >1.5hrs 5 | |
| PMO1 | How many days do you go for moderate-intensity activity, that causes small increases in breathing or heart rate such as brisk walking [or carrying light loads, cycling, swimming, volleyball]? | None 0  Once a month 1  2 to 3 times a month 2  Once a week 3  2 to 3 times a week 4  4 to 5 times a week 5  Every day 6 | |
| PMO2 | On average, how many minutes of moderate-intensity activity each day? | None 0 at least 10 mins 1  10 - 30mins 2  30mins - 1hr 3  1hr - 1.5hrs 4  >1.5hrs 5 | |
| PVI1 | How many days do you go for a vigorous-intensity activity that causes large increases in breathing or heart rate like [carrying or lifting heavy loads, digging or construction work , running or football or in a gym]? | None 0  Once a month 1  2 to 3 times a month 2  Once a week 3  2 to 3 times a week 4  4 to 5 times a week 5  Every day 6 | |
